# Hypergastrinemia and a duodenal ulcer caused by gastric duplication

**DOI:** 10.1186/s40792-016-0203-0

**Published:** 2016-07-27

**Authors:** Hideaki Tanaka, Kouji Masumoto, Takato Sasaki, Naoya Sakamoto, Chikashi Gotoh, Yasuhisa Urita, Toko Shinkai, Hajime Takayasu, Noriyuki Nakano, Masayuki Noguchi, Toyoichiro Kudo

**Affiliations:** 1 Department of Pediatric Surgery, Faculty of Medicine, University of Tsukuba, 1-1-1 Tennoudai, Tsukuba, Ibaraki Prefecture 305-8575 Japan; 2 Department of Pathology, Faculty of Medicine, University of Tsukuba, 1-1-1 Tennoudai, Tsukuba, Ibaraki Prefecture 305-8575 Japan; 3 Department of Pediatrics, Ibaraki Children’s Hospital, 3-3-1 Futabadai, Mito, Ibaraki Prefecture 311-4145 Japan

**Keywords:** Duplication cyst, Stomach, Hypergastrinemia, Duodenal ulcer, Child

## Abstract

**Background:**

Hypergastrinemia and the resultant peptic ulcer related to an enteric duplication has been quite rarely reported in the literature.

**Case presentation:**

We herein report the case of a 4-year-old girl who presented with hypergastrinemia and a duodenal ulcer at 2 years of age. She had been followed up with a proton pump inhibitor, which resulted in resolution of the ulcer; however, unexplained hypergastrinemia had continued. A cystic lesion at the antrum was discovered at 4 years of age, which we suspected to be a gastric duplication. After we resected the lesion, the hypergastrinemia resolved without recurrence of the duodenal ulcer. The histology was compatible with a gastric duplication, and the lumen was lined with antral mucosa that strongly stained positive for gastrin. We presumed that the antral mucosa inside the duplication in our case had no hydrogen ion feedback inhibition of gastrin release from gastrin cells and increased release of gastrin from the mucosa inside the duplication led to the duodenal ulcer. Only two cases have been reported in the literature that had hypergastrinemia related to enteric duplication.

**Conclusion:**

Gastric duplication should be included in the differential diagnosis of sustained hypergastrinemia in children.

## Background

Gastric duplications occur infrequently in children and most of the cases may become symptomatic as an abdominal mass, gastric outlet obstruction, or gastrointestinal hemorrhage [1]. We herein report an unusual pediatric case of gastric duplication that appeared to have caused hypergastrinemia and a duodenal ulcer.

## Case presentation

A 4-year-old girl with unexplained hypergastrinemia was referred to our department after abdominal ultrasonography detected a cystic lesion at her prepyloric antrum. When she first presented with a duodenal ulcer at 2 years of age, her serum gastrin levels had ranged between 700 and 1000 pg/ml. The imaging studies with a contrast-enhanced computed tomography (CT) scan had repeatedly revealed no tumor around the pancreas or duodenum suggestive of gastrinoma but revealed only a thickened wall of the duodenum potentially due to the initially severe duodenal ulcer. She had continuously taken a proton pump inhibitor (PPI) until the referral since the first presentation of the duodenal ulcer, which had relieved its symptoms such as epigastric pain.

**Fig. 1:**
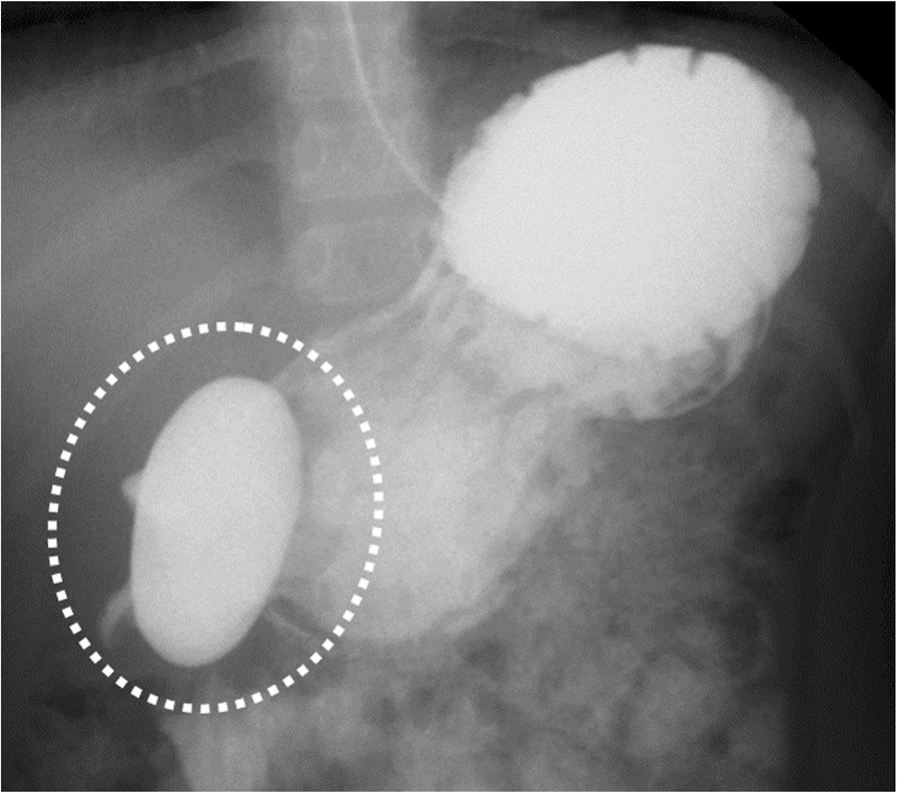
Upper gastrointestinal series revealed a 2.5 × 5 cm cystic lesion on the anterior wall of the antrum, communicating with the gastric lumen (*dotted circle*)

**Fig. 2:**
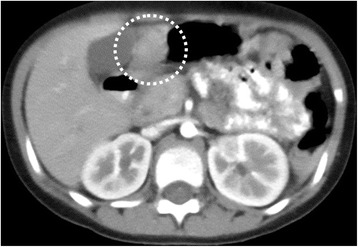
The abdominal contrast-enhanced CT with oral contrast detected a slightly enhanced solid mass at the antrum with no swallowed contrast inside (*dotted circle*)

**Fig. 3:**
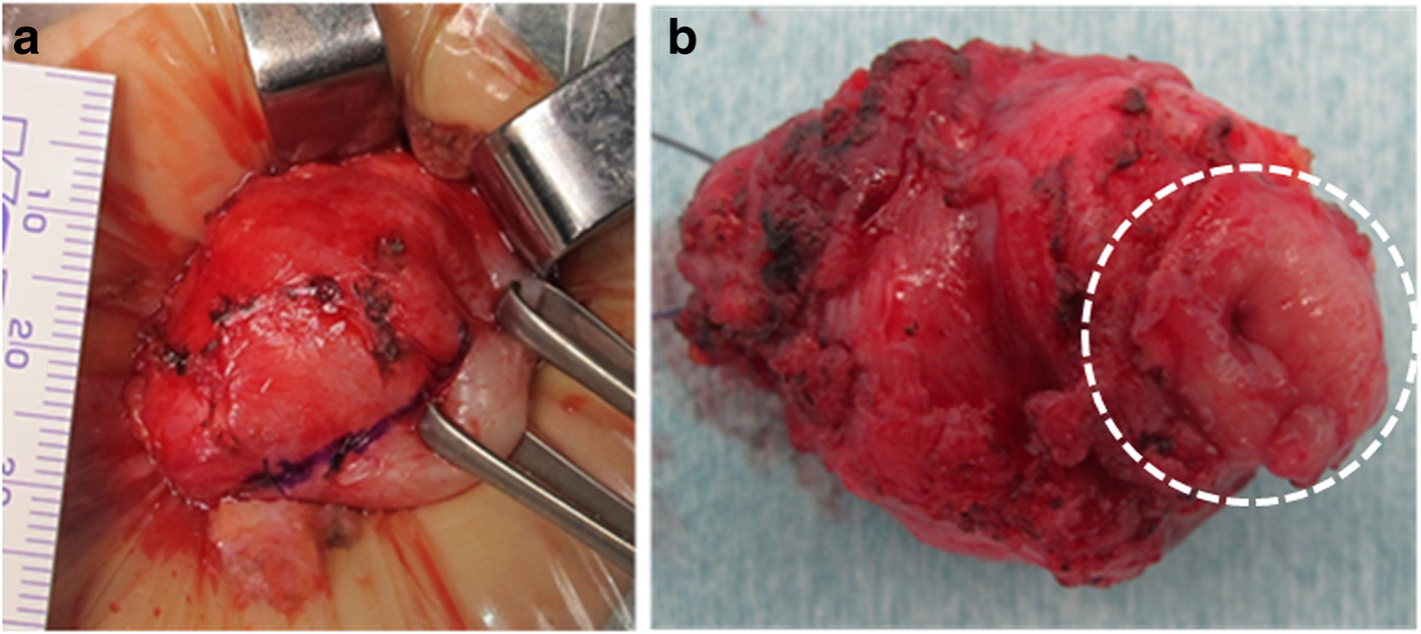
Intraoperative photographs. **a** A 2 × 3 cm protruded lesion was noted on the anterior wall of the gastric antrum. **b** The enucleated lesion had an opening to the gastric lumen (*dotted circle*)

**Fig. 4:**
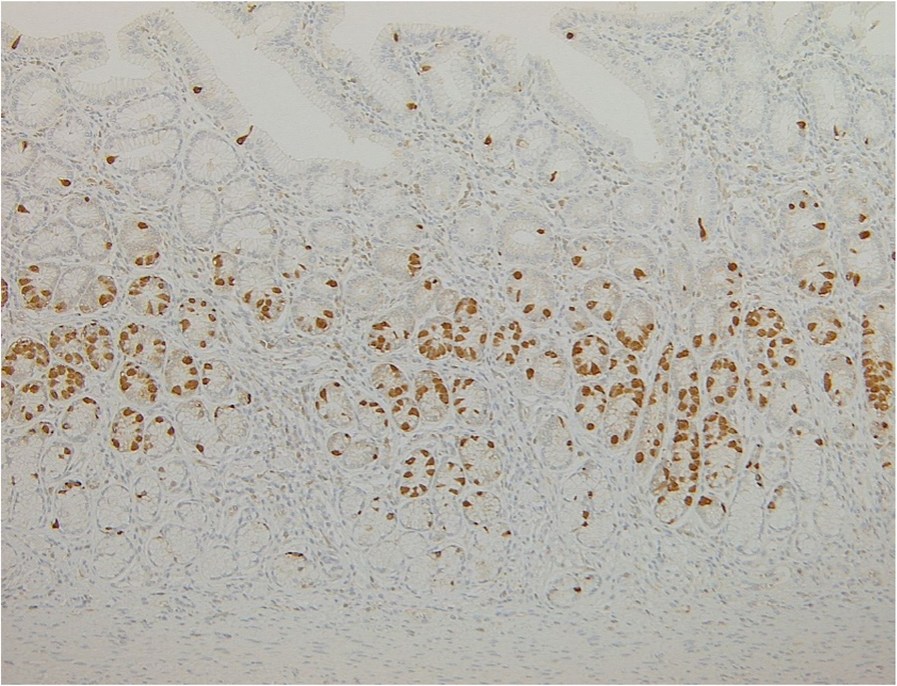
Immunohistochemical localization of gastrin demonstrated many positive cells at the glands of the antral mucosa inside the duplication

At the referral, the patient’s physical and mental development were normal for her age. She was afebrile, and other vital signs were also within the normal ranges. A physical examination revealed no particular symptoms. Her blood tests were unremarkable except for elevated serum gastrin (877 pg/ml, normal range 37–172 pg/ml). Upper gastrointestinal series revealed a 2.5 × 5 cm cystic lesion on the anterior wall of the antrum, communicating with the gastric lumen (Fig. 1), which was also detected as a solid mass with slight enhancement on abdominal contrast-enhanced CT (Fig. 2). Upper endoscopy showed a scar at the duodenal bulb, but no erosion or ulcers in the stomach or the duodenum, and we could not detect the opening of the lesion. A gastric tissue biopsy did not demonstrate *Helicobacter pylori*. According to these findings, we suspected gastric duplication. As we speculated that the lesion might have caused the duodenal ulcer in this patient, we proceeded with operative treatment for the lesion. The operation was performed with the umbilical approach. A 2 × 3 cm protruded lesion was observed on the anterior wall of the gastric antrum (Fig. 3a) and resected with the common muscular layer with the opening to the gastric lumen (Fig. 3b). A pathological examination was consistent with gastric duplication with antral mucosa, which strongly stained positive for gastrin (Fig. 4) and also contained heterotopic pancreas tissue. The postoperative course was uneventful, and the patient was discharged on the eleventh postoperative day (POD). Her serum gastrin level remained elevated after the operation (755 pg/ml on POD 7 and 727 pg/ml on POD 98) but started to decrease after PPI was switched to famotidine 3 months after the operation (228 pg/ml on POD 148 and 235 pg/ml on POD 291) and finally normalized after famotidine was stopped 10 months after the operation (151 pg/ml on POD 365 and 77 pg/ml on POD 461). She has remained entirely asymptomatic during a follow-up of 20 months.

### Discussion

Enteric duplications are ectopic cystic or tubular structures composed of smooth muscle surrounding the mucosa of the gastrointestinal tract and occur most commonly along the ileum, esophagus, or colon [2]. Gastric duplications are relatively rare in children, accounting for only 4–8 % of all enteral tract duplications [3]. They are usually spherical or tubular cysts in the greater curvature of the stomach and may not communicate with the gastric lumen [4]. Their presentations range from asymptomatic to an acute abdomen and include gastric outlet obstructions, pancreatitis, hemoptysis, gastrointestinal bleeding, and an ulcerated antral mass [5]; hypergastrinemia has been rarely reported [3] as one of the complications associated with a gastric duplication.

Normally, acid in the gastric lumen acts directly on the somatostatin cells (D cells) to stimulate the release of somatostatin, thereby preventing gastrin release from gastrin cells (G cells) by a paracrine mechanism. When gastric antral mucosa is isolated from its usual acidic environment, such as in cases of a retained gastric antrum following partial gastrectomy with Billroth II reconstruction, there is no hydrogen ion feedback inhibition of gastrin release, leading to hypergastrinemia followed by increased gastric acid production, recurrent ulceration, and bleeding [3]. We speculate that this same mechanism caused hypergastrinemia and the resultant peptic ulcer at the initial presentation in our case whose ectopic antral mucosa in the duplication barely communicated with her gastric lumen. If more gastric acidic juice comes into the duplication lumen through a larger opening and/or the amount of the G cells is less than that in our case, hypergastrinemia may not happen. A literature review revealed only one case of a gastric duplication [3] and another of a pancreatic duplication [2] presenting with hypergastrinemia. Stephen et al. [3] reported a female who presented with hematemesis and melena at 2 weeks of age. The possibility of gastrinoma was excluded using a secretin stimulation test and imaging studies; however, exploratory laparotomy could not detect any pathology, and thus, antrectomy and Billroth II reconstruction were performed. The pathological findings revealed gastroduodenal erosions and an antral duplication cyst with deep glands at the intracystic mucosa that stained positive for gastrin. Siddiqui et al. [2] reported the case of a 2-year-old girl who presented with failure to thrive and gastroesophageal reflux with continued low gastric pH levels. Portal and pancreatic venous sampling in that patient during the operation revealed elevated levels of gastrin to the pancreatic venous cascade, and CT demonstrated a cystic lesion in the head of the pancreas. The patient underwent local resection of the lesion, and a pathological study confirmed the duplication with gastric mucosa (immunostaining of gastrin was not performed).

Although the time course of the serum gastrin level of the latter patient was not described, that of the former patient normalized promptly after the operation. In contrast, the reason why hypergastrinemia in our case did not improve until PPI and famotidine were stopped may be explained by another mechanism related to the preoperative long-term use of PPI. A modest increase in the serum gastrin level in adult patients under a long-term treatment with acid-lowering compounds, especially PPI, has been reported [6]. PPI causes potent suppression of gastric acid secretion and leads to persistently high intragastric pH, and long-term PPI therapy has been reported to be associated with a significant increase in the G cell numbers and the ratio of G to D cells in the gastric antrum in children [7].

According to previous reports [6, 7], we speculate that the pathophysiology of our case was as follows: (1) the antral mucosa in the duplication was isolated from its typically acidic environment, which led to the lack of hydrogen ion feedback inhibition of gastrin release, and therefore, resultant hypergastrinemia caused the duodenal ulcer; (2) PPI suppressed gastric acid secretion and improved the duodenal ulcer, but the secretion of gastrin continued from the duplication for 2 years until it was removed, with an increase in the antral G cell numbers and the G to D cell ratio caused by the long-term use of PPI, thus additionally leading to hypergastrinemia; and (3) even after the removal of the duplication, hypergastrinemia continued until PPI and famotidine were stopped by the same mechanism.

## Conclusions

In conclusion, gastric duplication should be included in the differential diagnosis of sustained hypergastrinemia in children. In addition, clinicians should be aware that the long-term use of PPI may also cause hypergastrinemia.

## Abbreviations

CT: Computed tomography
PPI: Proton pump inhibitor
